# Atraumatic patellar prosthesis dislocation with patellar tendon injury following a total knee arthroplasty: a case report

**DOI:** 10.1186/1752-1947-4-11

**Published:** 2010-01-15

**Authors:** Vinay Kumar Singh, Pankaj Kumar Singh, Yashwant Singh, Alka Singh, Sadaf Javed, Murad Abdunabi

**Affiliations:** 1 Luton and Dunstable Hospitals NHS Foundation Trust, Lewsey Road, Luton, LU4 0DZ, UK; 2 Department of Neurovascular Surgery, Royal Hallamshire Hospital, Sheffield, UK; 3 Associate Specialist BRD Medical College, Gorakhpur, India; 4 ST2 Elderly Medicine, Bedford Hospital, Bedford, UK; 5 FY2 Luton and Dunstable Hospital, Luton, UK; 6 Luton and Dunstable Hospitals NHS Foundation Trust, Lewsey Road, Luton, LU4 0DZ, UK

## Abstract

**Introduction:**

Total knee arthroplasty is a well-established procedure with gratifying results. There is no consensus in the literature whether to routinely resurface the patella while performing total knee arthroplasty or not. Although an extremely rare occurrence in clinical practice, patellar prosthesis dislocation is a possible complication resulting from total knee arthroplasty.

**Case presentation:**

We report a rare case of atraumatic spontaneous dislocation of patellar prosthesis in a 63-year-old Caucasian man of British origin with patellar tendon injury. The patient was treated successfully through a revision of the patellar component and tendon repair. In two years follow-up the patient is asymptomatic with no sign of loosening of his patellar prosthesis.

**Conclusions:**

A thorough understanding of knee biomechanics is imperative in performing total knee arthroplasty in order to achieve a better functional outcome and to prevent early prosthetic failure.

## Introduction

Patellar resurfacing in total knee replacement remains a controversial area. It is debated whether the patella should be resurfaced in every case, should never be resurfaced or that resurfacing should be done only in certain circumstances [1]. The literature remains divided with plenty of evidence for both resurfacing and not resurfacing the patella as a routine practice. Resurfacing of the patella is associated with significant alteration of patellofemoral biomechanics. Isolated atraumatic dislocation of the patellar component rarely occurs after total knee arthroplasty (TKA) and injury to the patellar tendon due to dislocated patellar prosthesis has never been reported.

We report a case of patellar prosthetic dislocation in order to stress the need for a thorough understanding of patellofemoral biomechanics to avoid catastrophic early prosthetic failure in resurfaced patellar TKA.

## Case presentation

A 63-year-old Caucasian man of British origin who worked as a railway engineer presented to an orthopaedic surgery clinic in December 1998. Clinico-radiological examination confirmed severe bilateral osteoarthritis changes. He had more symptoms on his right knee than his left one. In June 1999 he underwent arthroscopic washout and debridement that confirmed severe tricompartmental degenerative changes.

He was managed non-surgically until May 2002 with anti-inflammatory medication and physiotherapy. His symptoms progressively deteriorated, which in turn necessitated TKA on his right knee. In September 2002, a subtotal knee replacement that spared the patella was performed on the patient. After the operation he complained of an anterior knee pain that was already interfering with his daily activities and his job. Failing any improvement in his symptoms he was offered patellar replacement which was performed in May 2003. A size 41 poly implant prosthesis was cemented in place, which was subsequently noted to have a normal intraoperative tracking.

**Figure 1:**
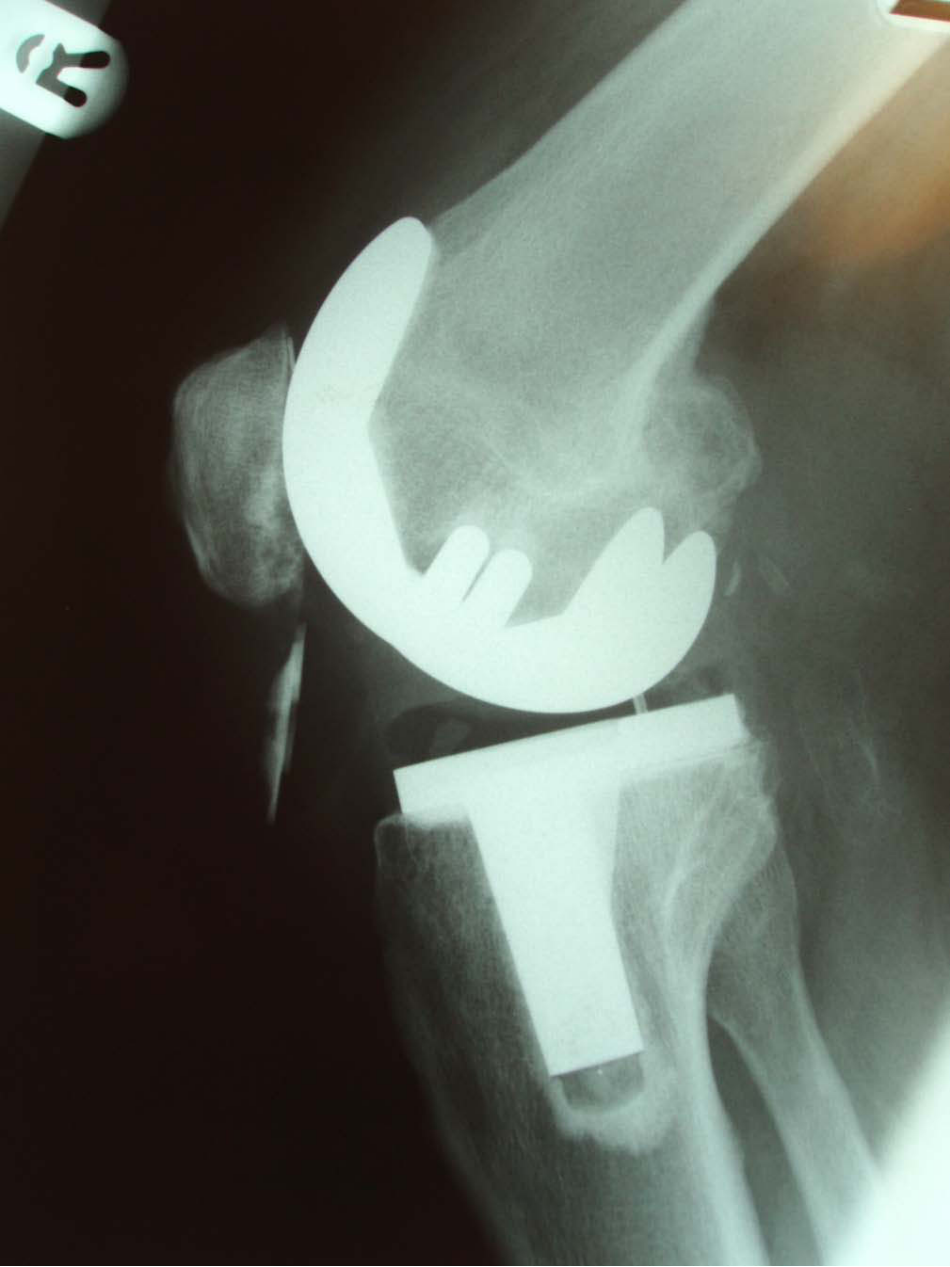
**Sagittal radiograph of the knee showing dislocated patellar prosthesis**.

The patient made a good recovery after the operation, but he complained of aching and clicking when flexing his knee from time to time. His symptoms improved and he was discharged from the clinic within one year. He presented again in January 2006 with a painful locked knee following a sudden flexion movement of his right knee. Radiographs taken in casualty showed that the prosthesis was dislodged and had displaced into the infrapatellar area (Figure 1). His blood test and inflammatory markers were unremarkable. Knee aspirate performed in theater to rule out infection was unremarkable.

**Figure 2:**
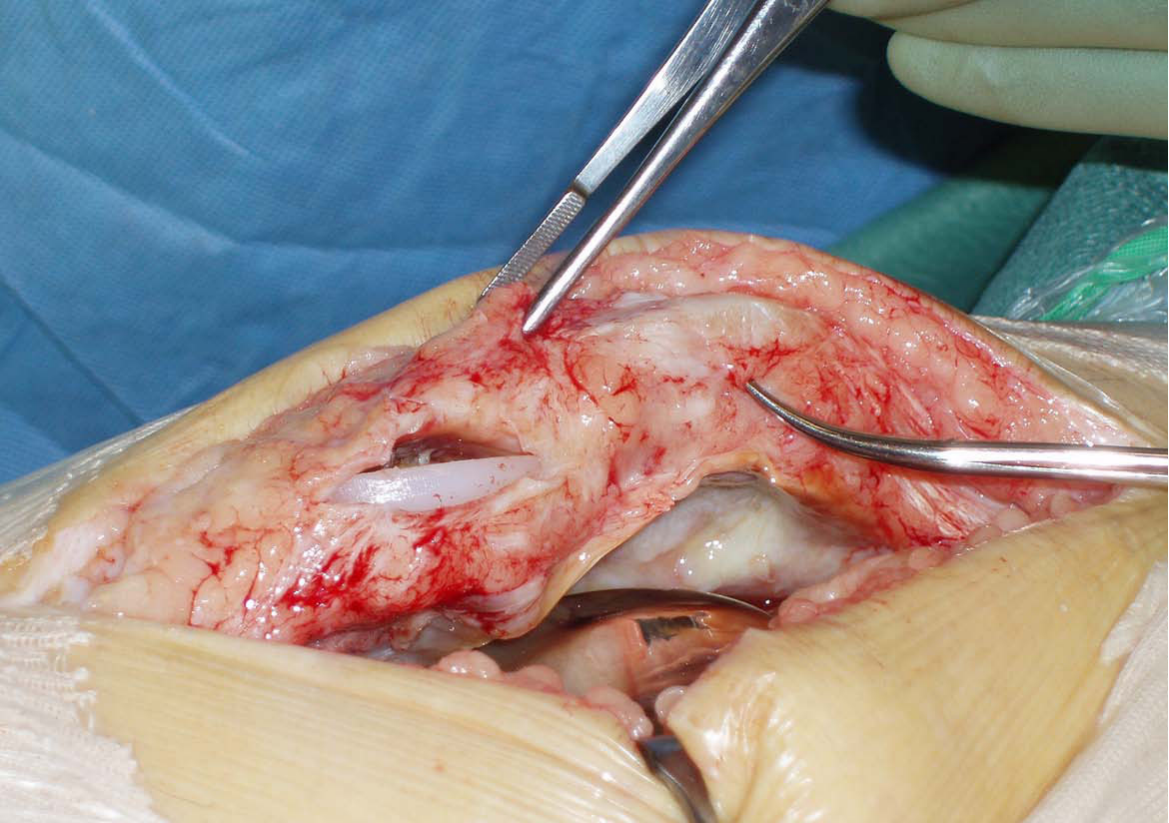
**Intraoperative picture showing dislocated patellar prosthesis in the patellar tendon**.

**Figure 3:**
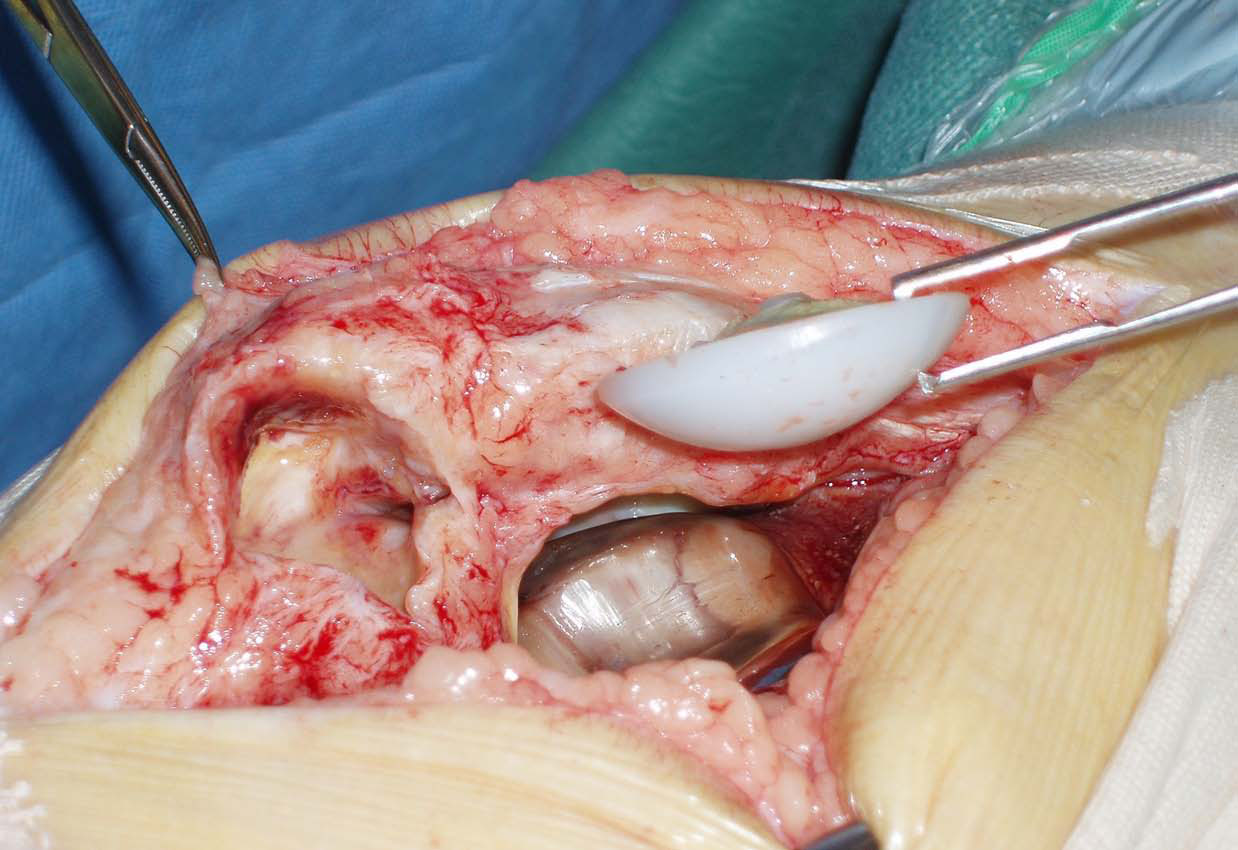
**Extracted patellar prosthesis with cavity in the patellar tendon**.

**Figure 4:**
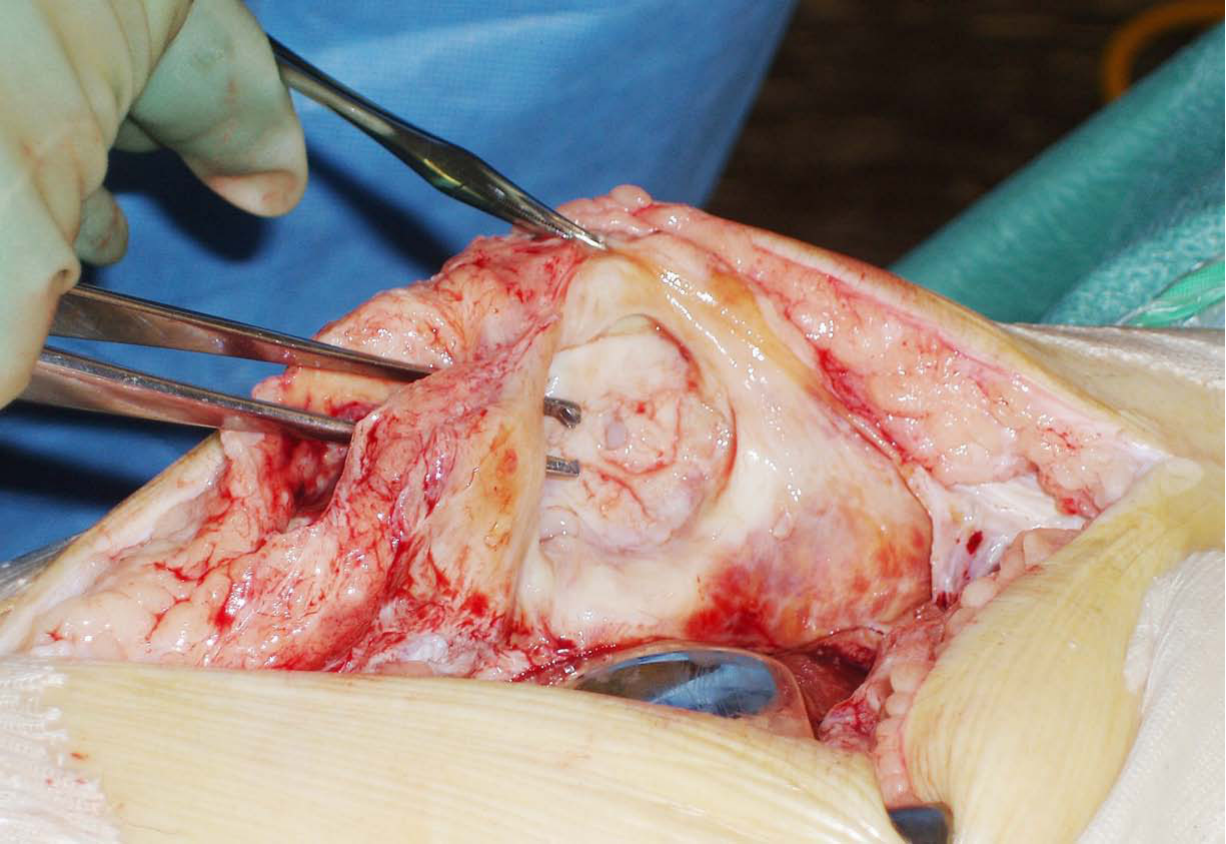
**Splitting of the patellar tendon due to dislocated patellar prosthesis**.

**Figure 5:**
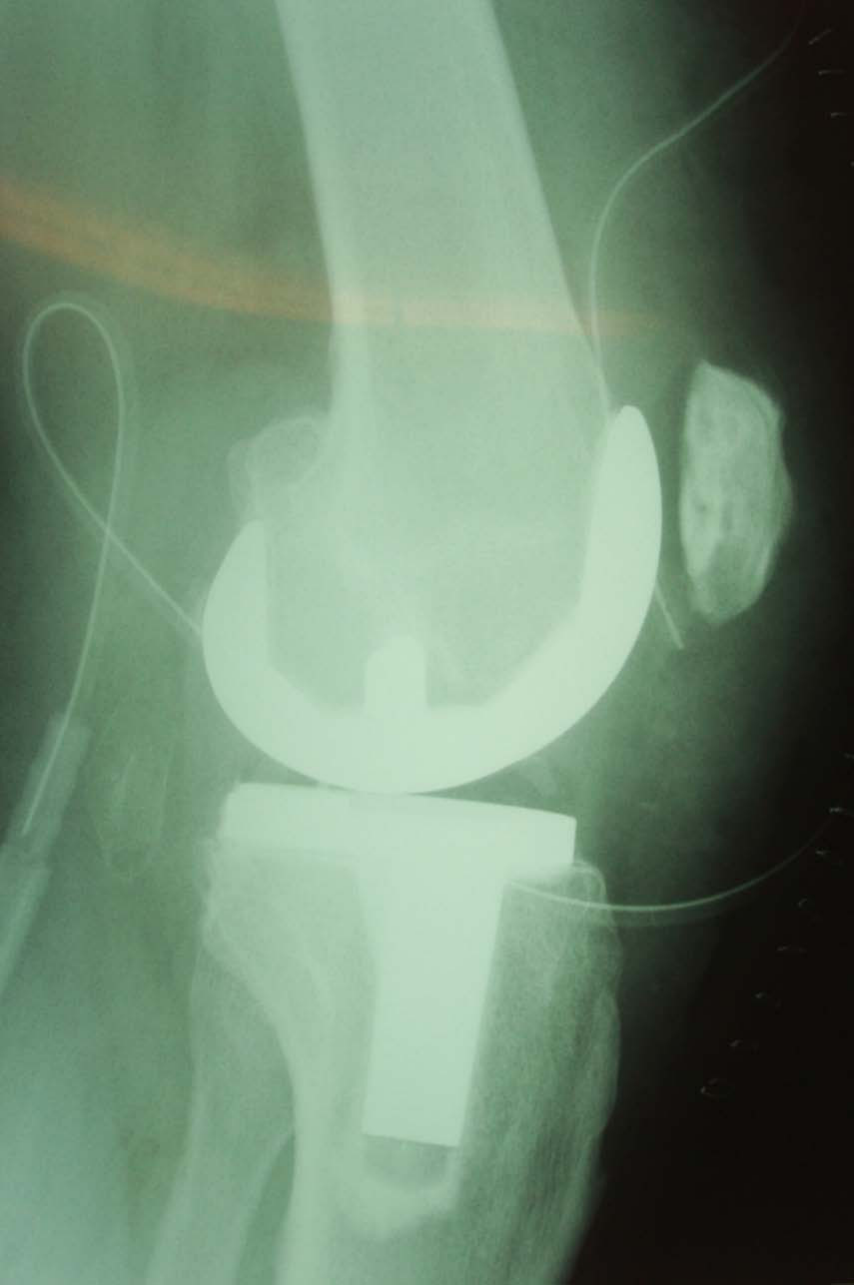
**Postoperative sagittal radiograph of the knee after revision of the patellar component**.

He underwent revision surgery of his right knee. Intraoperatively, the prosthesis was noted to have sheared off its fixation pegs and dislocated inferiorly, thus splitting the patellar tendon and forming a pocket in it (Figures 2, 3, 4). The patella was cleaned and the patellar cut was revised to 16 mm and the cemented 38-mm prosthetis was reimplanted with a good patellofemoral tracking (Figure 5). The patella tendon was cleaned and sutured using nylon suture. The postoperative period was uneventful and the patient was subsequently discharged from the hospital. He remains asymptomatic at two years follow-up and showed no evidence of loosening in his prosthesis.

## Discussion

Patella resurfacing remains a controversial issue among joint replacement surgeons. Recently published data from a 10-year prospective cohort study was unable to establish a significant difference between resurfaced and non-resurfaced groups [2].

There have been few case reports of intra- and extra-articular dislocation of patellar prostheses [3]. Previous reports have described dislocation but there was no damage to the tendon itself in any of the cases mentioned [1]. Furthermore, the shearing force evident in our patient was significant enough so as to have formed a pocket within the tendon and thus embedding the prosthesis itself.

Cadaveric studies estimate that although an increase in overall patella thickness may improve the effectiveness of the patella tendon at low flexion angles, a reduction in the range of movement and an increase in the risk of subluxation can occur [4]. Other studies show that patellofemoral contact stresses increase significantly when the patella is resurfaced. The distribution of force at the patellofemoral joint has almost always been altered as well [5,6].

Although most of the studies confirm that there is no difference between inserting and sparing the patellar prosthesis in TKA, the implantation of patellar prosthesis carries quite a significant morbidity and surgeons need to weigh the risks and benefits of such a decision. The operating surgeon needs to consider the biomechanics of the patellofemoral joint.

The original thickness of the patellar prosthesis component in our patient was 41 mm, thus raising the tension in the patellofemoral joint during movements involving high flexion angles. We postulate that this means that a sudden flexion has resulted in the shearing of the prosthesis through a force that was significant enough to penetrate the patellar tendon to a degree that is able to cause damage and require repair. Subclinical infection may also cause component loosing and dislocation. In our case, however, tests to rule out any possible infection all showed negative results. Following revision surgery, the new total thickness of the prosthesis was 25 mm, thereby resembling its natural dimension and restoring the forces closer to that of a normal knee.

## Conclusions

Dislocation of patellar prosthesis with injury to the patellar tendon is rare and has not been previously described in the literature. Patellar replacement is not a benign procedure, and it can lead to catastrophic failure that compromises the patient's condition if not performed correctly. A thorough understanding of patellofemoral joint biomechanics is crucial to create a close reproduction of the natural joint mechanics during surgery and to avoid early prosthetic loosing and failure.

## Abbreviations

TKA: total knee arthroplasty.

## Consent

Written informed consent was obtained from the patient for publication of this case report and any accompanying images. A copy of the written consent is available for review by the Editor-in-Chief of this journal.

## Competing interests

The authors declare that they have no competing interests.

## Authors' contributions

VKS collected clinical details and photographs pertinent to this case report. He also summarized the case history and prepared the first draft of the manuscript. PKS conducted literature review, designed and formatted the final manuscript YS: checked the manuscript for grammar, punctuation and style. AS assisted in conducting the literature search and in extracting necessary materials from the library and the internet. SJ: She verified the authenticity of the manuscript's scientific content. MA contributed in the preparation of electronic images used and in the electronic formatting of the manuscript.

Note: All authors have read and approved the final manuscript.
